# An Overview into Polyethylene Terephthalate (PET) Hydrolases and Efforts in Tailoring Enzymes for Improved Plastic Degradation

**DOI:** 10.3390/ijms232012644

**Published:** 2022-10-20

**Authors:** Nurul Fatin Syamimi Khairul Anuar, Fahrul Huyop, Ghani Ur-Rehman, Faizuan Abdullah, Yahaya M. Normi, Mohd Khalizan Sabullah, Roswanira Abdul Wahab

**Affiliations:** 1 Department of Biosciences, Faculty of Science, Universiti Teknologi Malaysia, Johor Bahru 81310, Malaysia; 2 Enzyme Technology and Green Synthesis Research Group, Department of Chemistry, Faculty of Science, Universiti Teknologi Malaysia, Johor Bahru 81310, Malaysia; 3 Advanced Membrane Technology Research Centre (AMTEC), Universiti Teknologi Malaysia, Johor Bahru 81310, Malaysia; 4 Department of Chemistry, Faculty of Science, Universiti Teknologi Malaysia, Johor Bahru 81310, Malaysia; 5 Enzyme and Microbial Technology Research Center, Faculty of Biotechnology and Biomolecular Sciences, Universiti Putra Malaysia, Serdang 43400, Malaysia; 6 Department of Cell and Molecular Biology, Faculty of Biotechnology and Biomolecular Sciences, Universiti Putra Malaysia, Serdang 43400, Malaysia; 7 Faculty of Science and Natural Resources, Universiti Malaysia Sabah, Kota Kinabalu 88400, Malaysia

**Keywords:** polyethylene terephthalate, plastic waste, biodegradation, PET hydrolases, recycling

## Abstract

Plastic or microplastic pollution is a global threat affecting ecosystems, with the current generation reaching as much as 400 metric tons per/year. Soil ecosystems comprising agricultural lands act as microplastics sinks, though the impact could be unexpectedly more far-reaching. This is troubling as most plastic forms, such as polyethylene terephthalate (PET), formed from polymerized terephthalic acid (TPA) and ethylene glycol (EG) monomers, are non-biodegradable environmental pollutants. The current approach to use mechanical, thermal, and chemical-based treatments to reduce PET waste remains cost-prohibitive and could potentially produce toxic secondary pollutants. Thus, better remediation methods must be developed to deal with plastic pollutants in marine and terrestrial environments. Enzymatic treatments could be a plausible avenue to overcome plastic pollutants, given the near-ambient conditions under which enzymes function without the need for chemicals. The discovery of several PET hydrolases, along with further modification of the enzymes, has considerably aided efforts to improve their ability to degrade the ester bond of PET. Hence, this review emphasizes PET-degrading microbial hydrolases and their contribution to alleviating environmental microplastics. Information on the molecular and degradation mechanisms of PET is also highlighted in this review, which might be useful in the future rational engineering of PET-hydrolyzing enzymes.

## 1. Introduction

Plastic was first invented in the 1860s and made from synthetic organic polymers, namely, fossil hydrocarbon derivatives. However, the demand and manufacturing of plastic began after the 1940s, topping most other manufactured materials, and became one of the fastest-growing global industries [1,2]. In fact, plastic has steadily substituted most natural materials, such as wood, metal, ceramic, stone, and leather [3], as the material is economical, flexible, and waterproof [4]. Plastic has become so important in today’s world that it is now considered one of the vital components of the textile, automotive, manufacturing, and packaging industries [5]. Plastic packaging accounts for over a third of all plastic polymers, representing 40% and 42% of the plastic demand in Europe and the USA [6]. The high demand for plastics is due to their excellent physical and chemical properties, such as their light weight, heat resistance, and high malleability. In addition to their transparency, hardness, and good tensile strength, plastics have become one of the most sought-after polymers for many applications [7].

The global shift from reusable to single-use containers is also a causal factor in the rise of plastic usage in the packaging sector [1]. However, the robust properties of plastic, which were once thought to be an advantage, are now the root of the steady rise in plastic waste in terrestrial and marine environments [8]. Plastics are persistent materials, given that the construction of monomers from fossil fuel-derived hydrocarbons takes up to ~1000 years to decompose naturally, thus accumulating in the environment [9]. Concomitantly, the global plastic output reached 348 million metric tons in 2017, with a worrying ~5% annual increase [10,11,12]. With current urbanization and population growth, the world plastic accumulation in the natural environment is projected to exceed 34 billion metric tons by 2050 [1,13]. More troubling, there are more reports on freshwater systems and terrestrial habitats increasingly polluted with synthetic fibers of plastic origin. Consequently, the resultant ubiquity of plastic waste in the environment has led to its use as a geological marker of the purported Anthropocene era [1].

The indiscriminate use of plastics and associated wastes with poor disposal substantially threatens wide-ranging environments of natural terrestrial, freshwater, and marine habitats [14,15]. Plastic waste debris, such as microplastics, is known to jeopardize animal life, the food chain, and human health [16,17,18,19,20]. The literature classifies the adverse effects of microplastic exposure to organisms into two, namely, physical and chemical effects. The physical effects are due to microplastics’ size, shape, and concentration, while the latter involve hazardous chemicals released from them [21,22,23]. Plastics disintegrate into microscopic particles (microplastics) with sizes of 5 mm to 1 μm once they reach the environment. The reported different types of microplastics include fragments, pellets, foams, rubber, and microbeads [24]. The damage of larger-sized plastics to ecology occurs when stray animals misinterpret them as food, while microscopic-sized plastics could pass into the water web and disrupt marine life [25,26]. Likewise, the buildup of microplastics could be hazardous to soil porosity, soil density, and the nutrient cycle, thereby adding to soil pollution [27]. In addition, microplastics enter the human food chain through contaminated foods, putting human health at risk, although recent studies show that microplastics have yet to impart severe long-term health effects on humans [21,22,23]. However, it has been suggested that microplastics could instigate oxidative stress in the body through reactive oxygen species during an inflammatory response, possibly leading to cytotoxicity. Microplastic reportedly could disrupt metabolism, energy balance, and immunity, upon transfer through food chains [17,23].

Most plastics produced today are manufactured from non-renewable petrochemicals derived from fossil fuels, natural gas, and coal. The types of plastics presently found in urban waste include polyethylene (PE), polyethylene terephthalate (PET), polypropylene (PP), high-density polyethylene (HDPE), polyvinyl chloride (PVC), polystyrene (PS), polyurethane (PUR), and low-density polyethylene (LDPE) [28,29]. Each of the aforementioned plastics is usually engineered to introduce specific physical properties, allowing their re-shaping into practically any form by rotation, injection, extrusion, compression, blowing, or thermoforming [30]. In the case of PET, DuPont developed the plastic in the mid-1940s, which is the most extensively used plastic in the packaging industry. The excellent mechanical, thermal, and chemical resistance and dimensional stabilities of PET are the main reason for its vast commercial utilization [5,31]. PET also has very low gas permeability compared to other polymers such as polyethylene, polystyrene, and polypropylene, making PET an ideal packaging material [32].

In today’s urban and industrialized society, plastics are now a necessity for the human population. Hence, efforts to recycle used plastic must be stepped up, given the hazardous nature of end-of-life PET waste to the environment. The future of plastic recycling should emphasize the circular economy approach, which integrates enzymatic processing to safeguard our environment for many years to come. Circular economy mainly focuses on preventing PET from becoming waste by diverting from the waste stream and redirecting it into beneficial economic activities [1,33]. Incorporating PET into the circular economy will be crucial to global efforts, particularly in combatting climate change and lowering the cost of PET production while requiring less water. Resorting to the circular economy could offer the advantages of creating small-scale initiatives for plastic recycling [34], which reduce the annual volume of plastics entering the oceans and greenhouse gas emissions.

PET is formed as a semi-aromatic polymer through polycondensation of terephthalic acid (TPA) with ethylene glycol (EG) or by the transesterification of dimethyl terephthalate with ethylene glycol [35]. Its noteworthy uses include disposable plastic bottles, food jars, and plastic film. PET production increased to 33 million metric tons in 2015 (Geyer et al., 2017) and currently represents 80% of total global plastic usage [8]. The persistence of PET waste in terrestrial and marine environments could harm or kill some organisms, as only a very low portion of this plastic is recycled to recover its original forms, such as TPA and EG [8,36]. Having said that, scientific research on PET should be geared toward sustainability by bioprospecting or developing more hydrolases that can cleave the ester linkages in the amorphous domain of PET to enable the bioremediation of PET [31], since various microorganisms naturally produce enzymes. Bio-based recycling can sustainably manage PET waste and degrade the produced monomers at the end of the process, yielding products with properties comparable to virgin PET that could be converted into high-value chemicals [37]. For example, Li et al. [38] established a value-added recycling strategy by reusing PET waste as an anti-stripping agent in asphalt mixtures. Another end-of-life management was attempted on the PET-degrading *Pseudomonas*, first discovered to metabolize ethylene glycol to produce polyhydroxy acids (PHAs). The acids were then modified into hydroxyalkanoyloxy-alkanoates (HAAs) for use as monomers in the chemo-catalytic synthesis of bio-PU [39]. With suitable enzyme tailoring technology, researchers should be developing and large-scale producing PET-degrading novel hydrolases specifically for plastic recycling and aim for technology-driven strategies to tackle the end-of-life PET crisis.

## 2. Disposal/Treatment of PET Wastes

Most plastic/PET waste is disposed of by landfilling or using physical treatment, such as incineration and chemical-based treatments. Nonetheless, these disposal methods have post-disposal environmental downsides such as releasing harmful pollutants and toxic by-products, secondary environmental pollution, major climate change, and threats to public health safety. With respect to these issues, recycling is a better alternative method to tackle the problem of massive accumulation of PET waste. The approach is more sustainable for treating PET waste, in which mechanical recycling is one of the most prevalent treatment forms for large-scale recycling of plastic solid waste [40]. Currently, PET waste recycling is far from efficient, yielding poorly recycled PET waste. The deficiency is due to mechanical stress such as segregation, grinding, crushing, re-extrusion, reprocessing, and photo-oxidation caused by the heat of fusion [7,41,42]. To date, there are no reports on microplastic generation during mechanical recycling [43].

**Figure 1:**
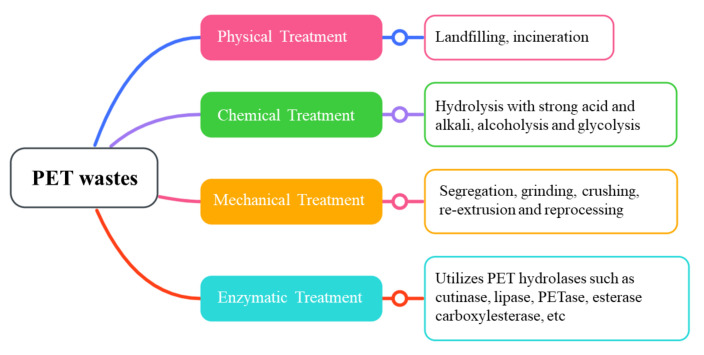
Recycling treatments to treat PET wastes.

The chemical-based recycling methods for PET recycling wastes involve the conversion of PET into lower molecular weight products [44]. These methods include hydrolysis (reaction with water using strong acids and alkalis) [45,46], alcoholysis (reaction with alcohol, ethanol, and methanol) [47,48], and glycolysis (reaction with some glycols, such as ethylene, or diethylene glycol) [49]. Although PET can be depolymerized by chemically assisted recycling, the process warrants the use of high temperatures and pressures, with the evolution of toxic by-products, thus incurring another issue related to serious secondary pollution. On that basis, the chemical-based recycling method is not recommended [5,9]. A summary of the treatments currently used to manage PET wastes is shown in Figure 1, while Table 1 lists the disadvantages and pollutants produced by these treatments.

## 3. Biodegradation of PET

The biological method to deal with PET wastes has emerged as a promising and eco-friendlier solution to meet the stringent environmental quality goals. The increasing awareness for improving the sustainability of plastics usage has been the driving factor in uncovering biologically safer methods to eradicate plastic waste that damages our environment [13]. This approach to deal with the abundance of plastic waste, i.e., PET, is the discovery of a variety of plastic-degrading enzymes from microbial sources. The degradation of PET through biological means is deemed a “green route” and provides a more sustainable approach to managing PET waste. Since ester bonds link PET monomers with a hydrolyzable functional group in their C-C backbone, the bioremediation of PET by specialized hydrolytic enzymes found in nature appears feasible. The literature revealed various microbial enzymes, including those from fungi and bacteria, with polyester-degrading mechanisms and could degrade synthetic and natural plastics [63]. Certain microorganisms were found to rely solely on plastics for carbon sources to survive and could thrive on plastic waste under optimal growth conditions [64,65].

One of the key metrics used to determine whether the plastic can be successfully biodegraded is the reduction in the molecular weight of the plastic monomer [66]. Initially, microorganisms colonize the plastic surface to reduce the polymer size before degrading it into its monomers, before they are taken up by the microbial cells. These monomeric units are further enzymatically degraded in the cells, using the monomers as carbon growth sources. The same concept applies during PET degradation, in which microorganisms attach to the surface of PET films to secrete extracellular PET hydrolases. Then, the hydrolases bind to the PET films, and the degradation process begins. PET hydrolases hydrolyze the ester bonds of PET for transformation to terephthalic acid (TPA) and ethylene glycol (EG), which then yields mono-(2-hydroxyethyl) terephthalate (MHET) and bis(2-hydroxyethyl) terephthalate (BHET), as incomplete hydrolysis products [37,67].

Scientists discovered that certain microorganisms had evolved novel biochemical pathways that produce specialized enzymes that remarkably break down PET [64,65,68,69]. For example, bacterial cutinases from the genus *Thermobifida* have been cloned and characterized as plastic-degrading enzymes, mainly because of their high degree of identity and similarity to PET hydrolase [70]. Certain fungal cutinase strains belonging to the genera *Saccharomonospora* [70], *Fusarium* [71], *Humicola* [72], and *Thermomonospora* [73] are the most studied for the hydrolytic degradation of polyester PET. Additionally, yeasts that belong to the genera *Candida* [74], *Pischia* [75], and *Aspergillus* [76] secrete lipases that hydrolyze PET [77]. Other bacterial species producing plastic-degrading esterases are *Bacillus* [78], *Clostridium* [79], and *Thermobifida* [80]. Several PET hydrolases from different microorganisms have been identified as crucial components for the biocatalytic recycling of plastic.

It is noteworthy to mention here that enzymatic recycling offers a greener avenue to depolymerizing and recycling PET waste [8,81]. This is because enzymatic PET recycling offers several benefits over chemical depolymerization. For instance, enzymatic PET biodegradation can be carried out under mild conditions, requiring less energy consumption [9]. Such a method has been successfully utilized in developing countries such as France and Japan [69], producing environmentally friendly and good-quality recycled plastic. Thus, the next subsection in this review article highlights the recent discovery of newly isolated enzymes from bacteria or fungi that could degrade PET. It is hoped that this review will help guide future research into further improving the enzymatic biodegradation of PET waste to alleviate its abundance in the environment.

## 4. PET-Degrading Enzymes for PET Degradation

PET hydrolases are a group of enzymes that include carboxylic ester hydrolases (EC 3.1.1) belonging to the α/β hydrolase family. This group of enzymes exhibits the ability to hydrolyze PET because of their water solubility. This class of enzymes has a low sequence identity but shares oddly similar folds [82]. The hydrolysis of PET begins when PET hydrolases consume the plastic polymer and break it down into simpler monomers, in order to adapt to the environment readily. This condition allows the microorganisms to assimilate the plastic monomers as major carbon sources, which are further metabolized into CO_2_, H_2_O, CH_4,_ and N_2_ [83]. To date, several types of hydrolases have been reported to be capable of degrading PET, namely cutinase (EC 3.1.1.74), lipase (EC 3.1.1.3), carboxylesterase (EC 3.1.1.1), PETase (EC 3.1.1.101), MHETase (EC 3.1.1.102), and esterase. Table 2 lists the various PET hydrolases from known microbial sources that hydrolyze PET.

The above-mentioned PET hydrolases share several common notable features, for instance, a solvent-accessible narrow active site, an active cleft having aromatic macromolecules, and an affinity for hydrophobic materials in the active cleft region [96]. However, Danso, Chow, and Streit [12] described PET hydrolases as enzymes with a low or moderate turnover rate toward PET substrate. The limited accessibility of the crystalline PET and its hydrophobicity, plus the enzymes’ temperature, pH, and specificity, are challenges for current PET hydrolases to efficaciously degrade PET, despite PET being highly available in the environment [5,7,97]. While PET is a non-biodegradable aromatic polyester, researchers have successfully identified several microorganisms producing unique hydrolases that could cleave the bonds in PET and initiate the biodegradation process [93]. The following subsections present an overview of recently reported studies on PET hydrolases. Further detailed studies are discussed in the following sections, accordingly.

### 4.1. Cutinase

**Figure 2:**
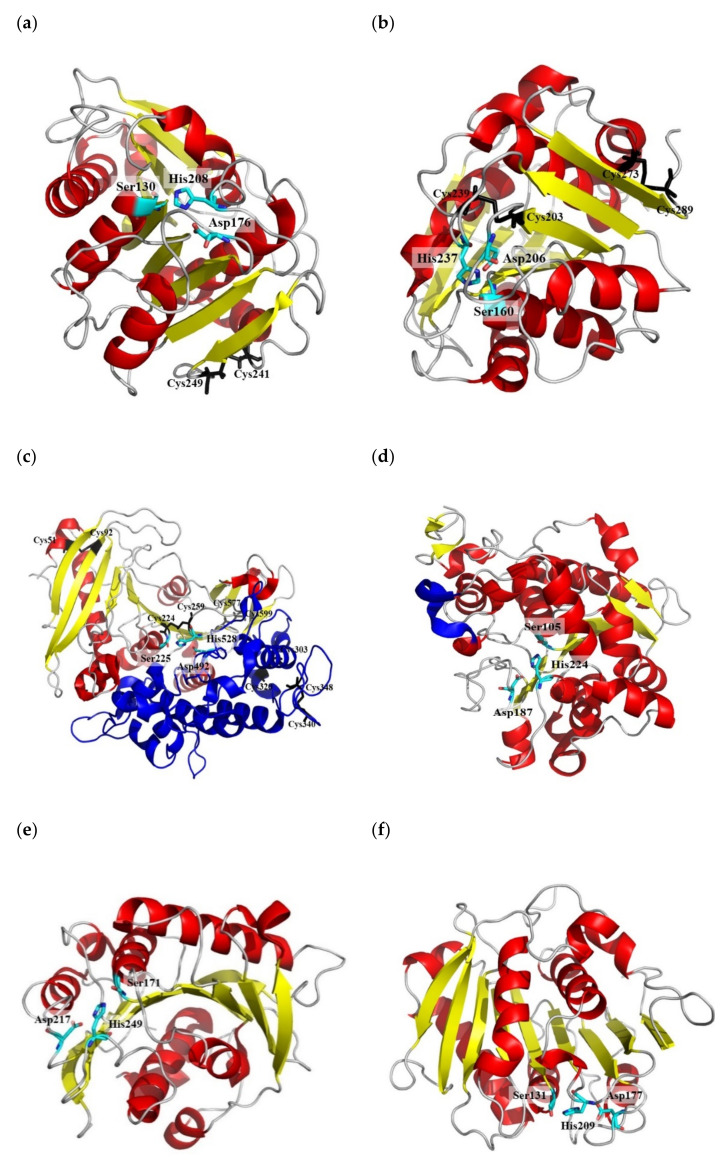
The three-dimensional protein folds of the different hydrolases that reportedly degrade PET (**a**) *T. fusca* cutinase structure with one disulfide bond (Cys241-Cys249) (PDB ID: 4CG3); (**b**) *I. sakaiensis Is*PETase with two disulfide bonds (Cys239-Cys203, Cys273-Cys289) (PDB ID: 5XJH); (**c**) *I. sakaiensis* MHETase with a large lid domain and five disulfide bonds (Cys51-Cys92, Cys224-Cys529, Cys303-Cys302, Cys340-Cys348, and Cys-577-Cys599) (PDB ID: 6QGA); (**d**) *C. antarctica* lipase with a α_5_-helix lid (PDB ID: 4K6G); (**e**) *P. aestusnigri* carboxylesterase (PDB ID: 6SBN); (**f**) *T. halotolerans* esterase (GenBank: AFA45122.1). Catalytic triad residues are highlighted in cyan, cysteine residues are presented by black sticks, and lid domains are in blue, respectively.

Among the many types of hydrolases, the enzyme cutinase (E.C 3.1.1.74) resembles a PETase the most. This enzyme belongs to the α/β hydrolase group, whose catalytic site architecture comprises a classical catalytic triad of Ser-His-Asp residues. The enzyme’s catalytic serine is uniquely not encased in an amphipathic loop, unlike lipase [98]. Cutinase is a promising enzyme for tailoring its protein structure to further enhance its ability to degrade PET, following its flexibility in hydrolyzing a broad range of ester bonds. Cutinase is also versatile in catalyzing esterification and transesterification reactions, which justifies its high usage as an industrial biocatalyst in the textile, detergent, and food industries [7,99]. Structurally, cutinase consists of a nine-stranded β-sheet, and eight α-helices with a disulfide bridge that lies between Cys241 and Cys249 (Figure 2a). Among the high-molecular-weight substrates of cutinase studied, cutin is one of the molecules that bind well with cutinase in its active site [96]. Cutinases have been isolated from plant pathogens, such as saprophytic microorganisms, which rely on cutin as the carbon source. The enzyme is also found in phytopathogenic microorganisms that break the cutin barrier to penetrate the host plants [7].

The first attempt to discover cutinases started about 50 years ago. Following that, several cutinases have been successfully isolated and characterized in the hopes of unraveling their structure–function relationships [100]. The literature has shown that cutinases were isolated in fungal and bacterial species, which primarily catalyze the breaking of ester bonds of cutin. Fungal cutinases have been reportedly isolated from *Penicillium citrinium* [101], *Humicola insolens* [72], *Fusarium solani pisi* [88], *Saccharomonospora viridis* [84], *Fusarium oxysporum* [102], *Aspergillus fumigatus* [103], and *Aspergillus nidulans* [104]. However, these cutinases only hydrolyze low-crystallinity PET. There are fewer reports on bacterial cutinases since the identity of their open reading frames has yet to be fully identified [98].

According to the literature, cutinases are known to degrade unnatural substrates comprising synthetic polyesters such as PET [102], polybutylene succinate [105], polycaprolactone [106], polystyrene (PS) [107], and polyethylene furanoate [108], along with other substrates, such as long-chain triacylglycerol or waxes [109]. While there are reports showing cutinases capable of hydrolyzing polylactic acid, such research is limited [110]. Meanwhile, a cutinase produced by *H. insolen* is more effective in degrading PET films than a cutinase produced by *T. cellulosilytica*, with a nearly complete enzymatic hydrolysis (97%) of a low-crystallinity (7%) PET film [72]. A study reported that recombinant cutinases of *Thermobifida cellulosilytica* DSM44535 (namely Thc_Cut1 and Thc_Cut2), and *Thermobifida fusca* DSM44342 (Thf42_Cut1) expressed in *E. coli* BL21-Gold(DE3), exhibited hydrolytic activity toward bis(benzoyloxyethyl)-terephthalate (3PET) and reduced crystallinity of PET film to 37%. At an optimum temperature of 50 °C, Thc_Cut1 released mono-(2-hydroxyethyl) terephthalic acid (MHET) and terephthalic acid (TPA). Conversely, Thc_Cut2 and Thf42_Cut1 degraded TPA as the major hydrolytic product [95]. In comparison, Thc_Cut2 of *T. cellulosilytica* exhibited lower hydrolysis efficiency than Thc_Cut1 due to the former’s hydrophobic surface properties. Moreover, amino acids on the surface of the enzyme are crucial for PET hydrolysis. Hence, by substituting selected Thc_Cut2 residues with those on Thc_Cut1 via site-directed mutagenesis, the hydrolytic efficiency of Thc_Cut2 of *T. cellulosilytica* might be improved [36]. Strategically placed substrate binding residues near the cutinase’s surface could facilitate the access for PET to the enzyme’s active site for catalysis.

In addition, cutinase exhibited maximum catalytic efficiency to hydrolyze p-nitrophenyl butyrate and p-nitrophenyl acetate [95,111], indicating that the enzyme binds preferably with shorter carbon chain substrates [7]. In terms of pH range, the majority of cutinases prefer neutral or alkaline pH environments. For instance, thermophilic bacteria *T. fusca* thrive best at pH values from 6.8−9.0, with pH 8.0 being the optimum and a preferred temperature from 50−55 °C [95]. The TfCut2 enzyme produced by *T. fusca* KW3 could hydrolyze PET films in an aqueous reaction system within an ultrafiltration membrane reactor. The ultrafiltration membrane enabled the above-said enzymatic reaction to progress for over 24 h at a 70% higher efficiency than batch hydrolysis [112]. The optimal hydrolytic condition was considerably different for fungal cutinase from *F. solani,* which works best at pH 7.5–10 [98,113], at 25 °C [113], 30 °C [98], and 40 °C [114]. Another example is the leaf and branch compost cutinase (LCC) which hydrolyzes different monoesters. The cutinase was isolated from the leaf–branch compost metagenome, and the hydrolase successfully degraded PET at pH 8.0 and 50 °C, displaying an enzyme activity of 12 mg/h/mg.

It is worth mentioning here that cutinases have distinctive characteristics when compared to lipases, as described by Gao, Pan, and Lian [96]. The catalytic triad (Ser–His–Asp) of cutinase is found at one end of the protein ellipsoid and is surrounded by loops [115,116]. Furthermore, cutinases form oxyanion holes before interacting with ligands, which is crucial in stabilizing anionic substrate complexes [117]. The oxyanion holes in cutinases reside at the active site, stabilizing the negative charge on the substrate ester or amide carbonyl oxygen during the formation of the tetrahedral intermediate to acyl transfer. This assembly is important for catalysis, commonly in serine proteases. As opposed to other lipases, cutinases have an oxyanion hole that is preformed, whereas lipases require structural rearrangement or binding to substrate in order to form one [99,118,119]. In contrast, PET hydrolysis activity is notably better in cutinases because the enzymes do not possess a hydrophobic lid structure. This means cutinases do not require interfacial activation, unlike lipases. The former’s active site catalytic serine is readily exposed to the solvent and behaves like interfacial activated lipase [120]. The exposed catalytic triad, Ser130–Asp176–His208, permits better acceptance of the hydrophobic PET substrate for hydrolysis. Hence, cutinases are more adept at accepting a wider range of substrates, which explains their ability to hydrolyze both soluble esters (substrate for esters) and insoluble triglycerides (the substrates for lipases). These enzymes also have numerous solvent-facing cation binding sites and catalyze short–medium-chain acyl esters with lengths up to C8−C10 [41]. Based on the above literature, it is apparent that cutinases are becoming one of the major groups of enzymes for PET hydrolysis.

### 4.2. IsPETase

Not long ago, Yoshida, Hiraga, Takehana, Taniguchi, Yamaji, Maeda, Toyohara, Miyamoto, Kimura, and Oda [65] reported a novel bacterial strain of *Ideonella sakaiensis* 201-F6, isolated from a plastic-bottle recycling factory in Sakai, Japan. This bacterium belongs to the genus *Ideonella* and the family *Comamonadaceae*. The bacterium produces a well-known PET hydrolase known as *Is*PETase, which hydrolyzes PET (ISF6_4831). A further structural analysis found that the *Is*PETase (EC 3.1.1.101) belongs to the α/β hydrolase superfamily, with a core structure of seven α-helices and nine β-strands of twisted central β-sheet conformation. The enzyme has a uniquely longer loop with three extra residues (Ser245, Asn246, and Gln247) than other homologous enzymes [31,97] (Figure 2b). It has been shown that the extended loops provide more space for the enzyme to bind with PET, whereas shorter loops inhibit the formation of subsites [49]. Compared to a cutinase, the high sequence identity of *Is*PETase regulated a conserved catalytic triad of Ser160–His237–Asp206, located in the loops behind β5, β7, and β8. In contrast, the serine residue in the catalytic triad of actinomycete cutinase is substituted with alanine in PETase [13,49]. Researchers have also discovered that *Is*PETase is active for extracellular PET hydrolysis and the subsequent intracellular pathway of PET-hydrolytic product degradation, confirmed by genetic and biochemical analyses [65,97].

Among all PET-degrading enzymes, *Is*PETase demonstrated its unique characteristics towards PET film at low temperatures, which caught the attention of many scientists. Liu et al. [121] described *Is*PETase as a homolog to actinomycete cutinase with 45−53% amino acid sequence identity, thus far. This is because, structurally, *Is*PETase has a broader open active-site architecture with an elongated substrate binding cleft consisting of subsite I and subsite II compared to cutinase. On subsite I, ester bonds are broken at a cleavage site, while on subsite II, Trp159 and Ser238 residues of *Is*PETase provide a passable space for the substrate to adhere [31]. Perspectively, a broader *Is*PETase active site increases the enzyme’s specificity for bulkier substrates such as PET, with no significant conformational changes upon ligand binding, compared to cutinase. As shown in Figure 2b, the catalytic residues of PETase (Ser160-Asp206-His237) reside on the protein’s surface, with a superficial groove sited above the nucleophilic serine. This is one of the reasons behind the ability of PETase to accommodate PET into its active site and efficiently degrade the compound. Notably, *Is*PETase possesses two disulfide bonds in its active site that could affect the enzyme’s thermal stability. The additional disulfide bond bridges the alpha and beta loops which contain the catalytic triad, whereas the previously studied cutinase has only one [31,36]. This structural evidence shows that the unique features in *Is*PETase are essential for efficient PET substrate binding. This information is useful for tailoring other enzymes in the α/β hydrolase superfamily, such as lipase and cutinase, to improve PET binding and degradation [97,122]. Considering the efficiency and specificity of *Is*PETase to hydrolyze PET, the enzyme is deemed a potential candidate for bio-based PET degradation strategies.

**Figure 3:**
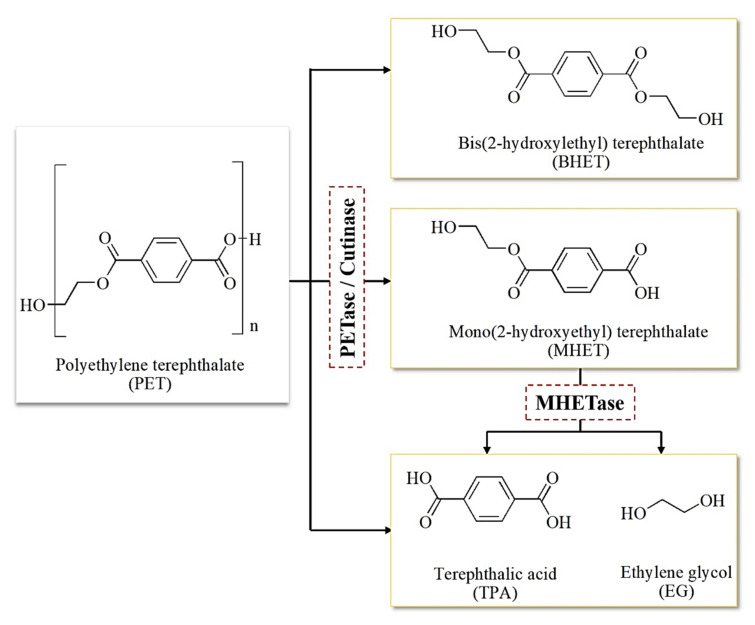
Schematic depiction of PET degradation catalyzed by PETase or cutinase. Polyethylene terephthalate (PET) is hydrolyzed by PETase/cutinase to produce bis(2-hydroxyethyl) terephthalate (BHET), mono(2-hydroxyethyl) terephthalate (MHET), terephthalic acid (TPA), and ethylene glycol (EG). MHET is hydrolyzed again by the second enzyme, MHETase, to yield terephthalic acid (TPA) and ethylene glycol (EG).

Compared to other previously reported PET-degrading homologs, the soil bacterium *I. sakaiensis* exhibited a relatively higher enzymatic activity, as high as 5.5- to 120-fold, than low-crystallinity cutinase, *Fusarium solani* cutinase, and *T. fusca* hydrolase at low temperature [93]. *Is*PETase also effectively degraded PET polyester under physiological conditions, specifically at 30 °C and pH 7.0, in which a 1.9% low-crystallinity PET film was used as a carbon and energy source [49,65]. Instead, cutinases typically degrade PET at high temperatures (50–70 °C), whereas PETase and MHETase prefer a lower degradation temperature (30 °C). The outcome seen here validates the ability of PETase to outperform other hydrolases to hydrolyze PET. Its novel discovery is a major breakthrough towards achieving high biodegradation efficiency of PET at ambient temperature. Generally, the *I. sakaiensis* bacterium secretes two enzymes, namely PETase (PET-degrading enzyme) and MHETase (MHET-digesting enzyme), whose role is to break down PET into simple and non-harmful monomers. The two enzymes work in synergy where PETase hydrolyzes PET polymer into mono(2-hydroxyethyl) terephthalic acid (MHET), producing TPA and bis(2-hydroxyethyl)-TPA as by-products. Further hydrolysis by the second enzyme, MHETase, produces two monomers, TPA and EG, which are then used as the bacterium’s food source [13,64,65]. Figure 3 illustrates the degradation of PET into different components catalyzed by cutinase or PETase. Besides PET, PETase also prefers p-nitrophenol (pNP)-linked aliphatic esters, the compounds used to measure lipase and cutinase activity.

### 4.3. MHETase

MHETase (EC 3.1.1.102) is another enzyme expressed by *I. sakaiensis* 201-F6, which works cooperatively with PETase to accommodate a two-enzyme system to completely degrade PET into TPA and EG monomers [65]. MHETase encompasses one of the α/β hydrolase family members showing good substrate specificity.

Comparable to other hydrolases, MHETase uses serine to execute a nucleophilic attack on the carbonyl (C=O) carbon [123]. A crystal structure of MHETase (PDB ID: 6QZ3) of *I. sakaiensis* 201-F6 was published by [124], revealing the architecture of the enzyme’s overall domain to be similar to feruloyl esterases. MHETase contains a large lid domain comprising ~240 amino acid residues (Tyr252–Ala469) situated between the β-strand (β_7_) and α-helix (α_17_) of the α/β hydrolase fold, which is crucial for the hydrolysis of MHET (Figure 2c). This lid domain consists partly of catalytic residues (Ser225, His528, and Asp492) and a Ca2+ binding site [125], increasing lid domain stability. This lid domain also exhibits 32.5% similarity with the closest structural homolog of feruloyl esterase (FaeB) found in *Aspergillus oryzae* (PDB ID: 3WMT) with several additional loops that distinguish it from FaeB [126]. MHETase is stable when disulfide bonds rigidify the catalytic triad. Nevertheless, there was a minor difference in the structure of MHETase, in which the enzyme is monomeric instead of having a dimeric structure [127]. The MHETase hydrolyzes optimally from pH 6.5–9.0 at 45 °C [126], with the enzyme reportedly capable of hydrolyzing non-hydrolyzable substrate analog (MHETA) or benzoic acid (Gao, Pan, and Lian [96]

Unlike PETase, PET hydrolysis by MHETase is not fully elucidated thus far due to limited studies on this enzyme. Both PETase and MHETase could hydrolyze PET efficiently at 30 °C. We will elaborate on an engineered MHETase recently shown to degrade PET as this structure has been extensively studied and, therefore, more hydrolase variants from MHETase are expected. The study successfully modified the active site of MHETase, producing new variants which show improved hydrolysis of PET. Most importantly, their findings provided valuable data on the molecular basis of product inhibition, improved activity against MHET, as well as renewed substrate specificity towards bis(2-hydroxyethyl) terephthalic acid (BHET) [96]. The engineered MHETase variants were shown to be promising candidates for cleaving materials closely related to the above-mentioned degraded products. Additionally, it has been discovered that the extracellularly generated MHETase may act as an exo-PETase to hydrolyze the synthesized PET pentamer. In addition to the engineered variant, an MHETase^R411K/S416A/F424I^ successfully demonstrated an increased BHET hydrolysis, which improved degradation activity against PET film [127].

### 4.4. Lipase

Lipases possess a close conformational similarity to the α/β hydrolase fold, and have a consensus motif of Gly–X1–Ser–X2–Gly lipases (EC 3.1.1.3). This is another class of hydrolases that has been explored for the enzymatic hydrolysis of PET due to the enzymes’ ability to degrade ester bonds [96]. The catalytic triad of lipases is made up of Ser–His–Asp residues, with serine (Ser) functioning as the nucleophile, histidine (His) as the basic residue, and aspartate (Asp) as the acidic residue [128,129].

The architecture of the canonical α/β hydrolase fold is built around a center, where lipases consist of eight parallel β-strands with one antiparallel β-strand (β_2_). The α-helices connect the strands of β3 and β8 to make up a complete protein structure in a lipase. Remarkably, the number of β-strands in lipases could be affected by the variations in the canonical fold, the presence of insertions, and the substrate binding domain architecture. This scenario hinders lipase’s nature and could lead to its catalytic promiscuity [130,131,132]. In addition, lipases hydrolyze long-chain (greater than C10) water-insoluble triglycerides preferentially, and their catalytic activity is distinguished by the interfacial activation mechanism compared to other hydrolases. Notably, a short polypeptide chain forms a lid on lipases which encases the active site. The lid regulates the exposure of the active site to solvents and substrates but also the development of an oxyanion hole during the nucleophilic assault on the substrate’s scissile bond [133,134].

It is pertinent to indicate that the lid’s presence over the active site entrance of lipases weakens substrate channeling to the substrate binding sites, which might reduce the hydrolysis activity, especially under unfavorable conditions. In addition, the lipase lid might block the entrance of the PET substrate into the tunnel, causing trajectory loss into the binding pocket of lipase, thus impeding catalysis. Thus, lipases require interfacial activation to induce catalysis for PET binding. As can be seen in Figure 2d, the catalytic residues (Ser105–Asp187–His224) are buried in the lipase core and are not facing the solvent, as opposed to cutinase and PETase, which have surface-groove active sites. Only certain lipase families could hydrolyze PET fibers, but not PET films [120]. Müller, Schrader, Profe, Dresler, and Deckwer [93] reported that the hydrolysis of aliphatic polyester nanoparticles (100 nm) by lipases was significantly faster than the polyester biofilm, in which a similar result was also observed for the aromatic polyester nanoparticles. The rapid degradation rate was thought to be caused by the poor crystallinity of polyester nanoparticles [135]. Since lipases are less likely to favor PET due to the lid structure that requires interfacial activation [37], further extensive mutational work is required to engineer the enzymes’ binding pockets. This enzyme tailoring strategy needs to emphasize improving the accessibility for PET entry and the correction trajectory/orientation to properly bind with the active site residues. This strategy can improve substrate specificity and enhance the enzyme’s efficiency in degrading PET.

Gupta et al. [136] reported that lipases showed improved degradation of PET textiles by improving their physiochemical characteristics such as wettability, dye-ability, and absorbency. Several fungal and bacterial organisms were reported to produce lipase, as whole-cell catalysts for PET digestion, such as *Candida antarctica* [137], *Triticum aestivum* and *Burkholderia* spp. [138], *Thermomyces lanuginosus* [85], etc. These bacterial lipases were observably 50-fold more efficient at bioconverting PET into MHET than fungal lipase, which requires the further addition of plasticizers to convert the PET into MHET [85]. In 2005, purified *Thermobifida fusca* lipase (TfH) hydrolyzed ~40–50% PET films at 55 °C within three weeks (Müller, Schrader, Profe, Dresler and Deckwer [93]. Lipase from *Thermomyces lanuginosus* was discovered by Eberl, Heumann, Brückner, Araujo, Cavaco-Paulo, Kaufmann, Kroutil, and Guebitz [85] to be capable of hydrolyzing PET. The enzyme afforded appreciable quantities of hydrolysis products from the model substrate PET in the presence of surface-active molecules, which promoted the lipase’s interfacial activation. Meanwhile, a bacterial consortium of three *Pseudomonas* spp. and two *Bacillus* spp., acquired from soil samples from locations polluted with petroleum products in Texas, were adept in degrading PET plastic at 30 °C after six weeks of incubation [139].

Another type of lipase from *Candida cylindracea* (CcL) and *Pseudomonas* sp. (PsL) effectively degraded PET nanoparticles at 30 °C and pH 7.0 [140]. Correspondingly, Wang, Lu, Jönsson, and Hong [76] apply BHET/TPA-induced lipase from *Aspergillus oryzae* for the hydrolysis of PET. Lipase B was effective because of its superficial catalytic site, which could interact with the substrate even without a hydrophobic surface, compared to other existing lipases [141]. The study used the combination of lipase B from *C. antarctica* (CALB) and *H. insolens* (HiC) to effectively hydrolyze PET to TPA. It is suggested that HiC performed better with PET hydrolysis; however, the enzyme demonstrated limited ability to convert MHET (one of the intermediates of PET hydrolysis) to TPA [74,142]. Conversely, CALB could transform MHET into TPA but exhibited a lower efficiency when used alone to hydrolyze PET [74]. The two lipases were seen to work synergistically to enhance PET hydrolysis following their complementary properties both in catalysis patterns and substrate specificity [8,143]. That said, the findings showed that the enzymes make ideal bioagents for the future biodegradation of plastics.

### 4.5. Carboxylesterase

Carboxylesterase (EC 3.1.1.1) is a ubiquitous enzyme that has been identified in both prokaryotes and eukaryotes. Structurally, carboxylesterase adopts a highly conserved protein architecture of α/β hydrolase folding, with eight stranded β-sheets, surrounded by α-helices on both sides and connecting loops [144] (Figure 2e). Carboxylesterase has a broad substrate specificity due to its open, active site and a distinctive binding pocket that permits binding with a wide-ranging substrate [145]. This enzyme accommodates a catalytic triad composed of serine, glutamic acid, and histidine, which reportedly show the ability to hydrolyze PET polymers. As opposed to lipases, carboxylesterases show distinctive criteria that discern both enzymes. Carboxylesterases hydrolyze water-soluble and short-chain acylglycerols (*<*10 carbon atoms), whereas lipases prefer water-insoluble long-chain triglycerides (*>*10 carbon atoms) [146,147]. Carboxylesterases also do not require interfacial activation for catalysis and do not involve any lipid/water contact for the active site to function efficiently.

An actinomycete thermophilic *T. fusca* KW3 (TfCa) was shown to produce a carboxylesterase that could hydrolyze PET fibers at 50 °C and pH 8.0 while retaining 37% of its activity after 96 h of incubation [91]. It has been shown that TfCa exhibited the typical substrate specificity of a carboxylesterase as it displayed favorable specificity, mainly towards short- and medium-chain-length fatty acyl esters of p-nitrophenol. The thermostable TfCa was previously employed to catalyze the modification of synthetic aromatic polymers and oligomers [91]. In 2020, a novel polyester-degrading carboxylesterase was discovered as part of the genome of *Pseudomonas aestusnigri*, a mesophilic marine bacterium [89]. This carboxylesterase, PE-H, was identified as a PET hydrolase enzyme (type IIa) and featured canonical α/β hydrolase folding similar to known polyesterases. PE-H reportedly hydrolyzed amorphous PET film at 30 °C, yielding an intermediate product of MHET. Despite its inability to hydrolyze PET bottle films, the wild-type PE-H enzyme was subsequently rationally mutated to give forth variant PE-H Y250S, showing improved hydrolytic activity toward PET bottles [89].

### 4.6. Esterase

Esterase exists in almost all living organisms, facilitating the cleavage of ester bonds (short-chain acyl ester) in PET monomers, producing surface-modified PET fibers [5,7]. Structural studies show that esterases have a classical α/β hydrolase folding structure showing distinctive central β-sheets surrounded by α-helices. For instance, the three-dimensional structure of hyperthermophilic esterase (EstE1) isolated from a fosmid metagenomic DNA library of a thermal environment comprised eight α-helices and eight β-strands [148]. Similar to serine proteases, the catalytic triad of an esterase comprises Ser–Asp–His residues [149] (Figure 2f). Instead of acting as hydrolases in plants, esterases commonly catalyze the biosynthesis of polyesters in the cuticle matrix rather than through hydrolysis reactions [150]. However, esterase activity appears to be limited to short-chain acyl esters compared to those hydrolyzed by lipases. There have not been many reports on the hydrolysis of hydrophobic PET by the enzyme. The first degradation of PET by esterases was reported for *Bacillus* and *Nocardia* [151]. Ribitsch, Heumann, Trotscha, Herrero Acero, Greimel, Leber, Birner-Gruenberger, Deller, Eiteljoerg, and Remler [78] employed *Bacillus subtilis* p-nitrobenzylesterase (BsEstB) to hydrolyze PET into TPA and mono(2-hydroxyethyl) (MHET) TPA using bis(benzoyloxyethyl) terephthalate (3PET) as a substrate, with the optimum condition occurring at pH 7.0 and 37 °C. Ribitsch, Acero, Greimel, Eiteljoerg, Trotscha, Freddi, Schwab, and Guebitz [94] also described that a recombinant esterase from *T. halotolerans* (Thh_Est) degraded PET into terephthalic acid (TA) and mono(2-hydroxyethyl) (MHET). The *Thermobifida* esterase *(*Thh_Est) revealed active surface hydrolysis for PET polyester, and its impact was comparable to that of cutinase from the same genus [80]. Similarly, Kawai, Oda, Tamashiro, Waku, Tanaka, Yamamoto, Mizushima, Miyakawa, and Tanokura [84] reported a recombinant thermostabilized polyesterase from *Saccharomonospora viridis* AHK190PET showing enhanced PET-hydrolyzing activity after calcium ions were added to the reaction mixture.

Overall, it can be proposed that cutinases, especially actinomycete cutinases, are the key enzymes that could catalyze PET hydrolysis, compared to other PET hydrolases mentioned in previous sections. This is because of the broader substrate specificity of Actinomycete cutinases compared to lipases, with the former showing a higher capacity for hydrolyzing a wider range of polyester fibers. Cutinases contain an open, active site surrounded by hydrophobic amino acid residues, and lipases comprise a lid covering the active site, which reduces their ability to degrade PET [41]. The unique characteristic of cutinases gives easy accessibility of PET substrates to bind with the enzyme to catalyze efficient hydrolysis of PET [122]. Given the above facts, the recently isolated cutinase-like enzyme, or PETase from *I. sakaiensis*, is truly a promising candidate for sustainable biodegradation strategies to reduce plastic contaminants in the environment.

## 5. Conclusions and Future Prospects

The accumulation of plastics in our seas, oceans, and landfills raises serious concerns about their possible environmental impact. In fact, the COVID-19 pandemic exacerbates this issue as it hampered the implementation of plastic reduction policies [36,152], with an increased burden of plastic medical wastes from personal protective equipment such as masks and gloves. This two-year pandemic era also saw single-use plastics and food packaging being used and discarded in unprecedented amounts by the healthcare sector and the general population [153]. These pandemic-related plastic wastes, unfortunately, make their way into the environment and oceans, adversely affecting marine wildlife, and potentially harming or killing them. This gives further challenges to the authorities in curbing the spread of the virus if the plastic wastes are not disposed of properly [154]. While conventional methods, such as landfilling, incineration, as well as mechanical and chemical recycling of PET wastes, are in place to tackle this issue, these methods have challenges of their own and produce undesirable by-products, which could pollute marine and terrestrial environments [155].

Since not all plastics can be recycled using the recycling methods mentioned above due to their costly production, there should be increased efforts by the government and manufacturing sectors to employ bio-based and biodegradable alternatives to remove PET from the environment. These programs should be a part of the solution to combat plastic pollution, with the integration of other strategies which generate revenue, such as energy generation, from enzyme-assisted PET degradation. The return on investment from energy generation could be used to offset the high cost of the bio-based PET degradation strategy. Consequently, increased concerted efforts to tailor existing enzymes isolated from various environments could increase plastic degradation, complementing and enhancing plastic recycling processes. This is because the physicochemical treatments are inadequate to remove PET and other plastic materials. Thus, these microplastic particles remain prevalent in our oceans, seas, and terrestrial environments. That said, the efficacy of certain cutinases in hydrolyzing PET could be used on a larger scale to treat plastic- and microplastic-contaminated environments. The discovery of plastic-degrading microorganisms and enzymes sparks new hope in their use for recycling and degrading PET; therefore, more effort should be put into developing more efficient bioreactor systems to degrade PET.

Microorganisms that degrade plastic are living bioreactors performing enzymatic hydrolysis, typically taking over 48 h to complete. Increasing the reactor’s enzyme loading could shorten the operational time frame. Scaling up the enzyme reaction for industrial applications would be another challenge due to the high production costs of the enzymes [156]. This issue could be solved with articulately well-designed bioreactors that support the conditions to maintain enzymes at their peak performance while negating the other issues that might complicate PET degradation, such as costs, the need for a large space, and inconsistent bioreactor performance. Bioreactor design requires considerations of many aspects, viz., the type and size of the bioreactor, type of inoculum system, incubation period, and PET concentration, all of which would change when the reactions use different enzymes. Recent study elucidates that the engineered *Pseudomonas putida* could degrade BHET into TPA and EG, and convert TPA into 15.1 g/L of β-ketoadipic acid (βKA) at 76% molar yield in 3 L fed-batch bioreactors within 48 h of cultivation in a bioreactor [157]. Waste PET, therefore, can be upcycled through this biological conversion. 

Likewise, protein engineering procedures could be used to augment enzyme-encoding genes or genome mining to discover new PET hydrolase genes that meet industrial needs. With a better understanding of PET hydrolases’ structural features, the substrate specificity of similar hydrolases, such as lipase and esterase, could be engineered. The focus should be on expanding their substrate specificity to break down plastic polymers with similar structures or having ester linkages. Significantly, additional study into the molecular function of PET hydrolases, by resolving their crystal structures, would also add to current knowledge in tailoring current enzymes for safer bioremediation of environmental PET. Computer advancements could aid computational and structural biologists, biochemistry researchers, and material scientists in further exploring this avenue [13], therefore possibly addressing plastic pollution for a safer and sustainable future.

Despite this, studies on tailoring novel PET hydrolases from existing isolated microbial enzymes to resolve the widespread microplastic pollution remain a significant challenge to many researchers. Isolating highly functional plastic-degrading microbes or enzymes is time-consuming and often does not yield sufficiently effective enzymes or microbes to degrade microplastics in the environment. Even so, it is nearly impossible for the global economy to do away with new plastic products. The world is not fully ready to embrace a plastic-less world, mainly because of the versatility and robustness of plastic materials. Since most natural enzymes do not meet the critical needs of industrial applications, they should be engineered into better, highly functional variants. The approach of enzyme tailoring for increased PET degradation efficacy, for instance, protein engineering, structure-guided mutagenesis, and rational enzyme modification, may prove to be a more expedient way forward. Just as new and powerful drugs are designed by computational means, the same concept is applicable for tweaking the three-dimensional structures of existing enzymes having similar α/β hydrolase folding as that of known PETases. It is no doubt a monumental feat, but researchers’ current efforts should focus on capitalizing on current in silico enzyme tailoring software and plugins. Moreover, computer hardware and software have considerably advanced to better screen and predict the outcome of novel-constructed enzymes before further empirical research is carried out.

New advances in protein engineering permit the design of novel microbial enzyme consortia with improved stability, catalytic activity, substrate specificity, and hydrolytic activity toward PET. This approach could quickly alleviate the ever-increasing microplastics and plastics in the environment [124,158,159], given the long-life expectancy of plastics [30]. This reduces the microplastic bioaccumulation in the food chain, thereby reducing medical costs while providing a safer, more cost-effective measure of environmental clean-up. All in all, breakthrough strategies in enzyme tailoring for enhancing PET biodegradation would prodigiously contribute to the plastic recycling industry while ensuring a better and plastic-contaminant-free future.

## Figures and Tables

**Table 1:** Disadvantages of three different treatment methods and toxic pollutants produced during these treatments.

Treatment Methods	Pollutants	Disadvantages	References
Physical process	Incineration		
	Emission of hydrocarbon oxides Sulfur dioxide Ammonia Corrosive organic acids Dioxins Furans Mercury Polychlorinated biphenyls	Requires high temperatures and pressures Releases toxic pollutants, heavy metals, and combustion products Serious secondary pollution Public hazard	[6,50,51]
	Landfilling		
	Heavy metals (cadium, lead, benzene, and dioxin) Leachate Toxins Greenhouse gases (methane, carbon dioxide)	Groundwater pollution Releases toxic pollutants and heavy metals Release of greenhouse gases resulting in climate changes Disrupts natural enzyme production by soil microbes, rendering the soil infertile Risk to human and animal health Needs a large ground space to bury the waste	[52,53,54,55]
Chemical process	Barium Benzene Cadmium Lead Selenium Toluene Mercury Arsenic Dioxins Ethyl benzene Acetaldehyde Formaldehyde Hydrochloric acid Methanol Hexane	Expensive operation Requires high energy, chemicals, and toxic reagents Requires high temperatures and pressures Large carbon footprint Evolution of toxic by-products Serious secondary pollutions Mainly limited to condensation polymers	[5,9,46,56]
Mechanical process	No toxic pollutants produced because the plastics are mechanically recycled	Requires detailed sorting/pre-treatment before the recycling process Relatively inexpensive Heterogeneity of solid waste Degradation of mechanical properties of plastics Difficult to recover large amounts of targeted plastics from mixed municipal plastic waste Inconsistent quality products Poses toxicological risk to aquatic ecosystems	[43,56,57,58,59,60,61,62]

**Table 2:** Biochemically characterized known microbial enzymes linked to polyethylene terephthalate (PET) biodegradation.

Enzyme	Microbial Sources	GenBank or PDB Code	PET Used (Substrate)	Degradation Temperature (°C)	Degradation Product	References
BsEstB	*Bacillus subtilis 4P3-11*	ADH43200.1	3PET	40–45	TPA, BA, MHET	[78]
CALB	*Candida antarctica*	P41365.1	Low-crystallinity and biaxially oriented PET films	50–60	TPA, BHET, MHET	[74]
Cut190 (S226P/R228S)	*Saccharomonospora viridis AHK190*	BAO42836.1	Amorphous PET film and package-grade PET	60–65	TPA, MHET	[84]
Cbotu_EstA	*Clostridium botulinum ATCC3502*	KP859619	PET film	50	TPA, MHET	[79]
FsC	*Fusarium solani pisi*	1CEX	Low-crystallinity PET (7%)	30–60	5% lcPET weight loss	[65,71,72,85,86]
HiC	*Humicola insolens*	4OYY	Low-crystallinity PET (7%) Crystallinity PET (35%)	30–85	97 ± 3% weight loss	[72]
*Is*PETase	*Ideonella sakaiensis 201-F6*	GAP38373.1	Low-crystallinity PET (1.9%), bottle-grade high crystallinity	20–45	TPA, MHET, EG	[65]
LCC	*Uncultured bacterium from leaf-cutinase branch compost metagenome*	AEV21261.1	Amorphous PET film	50–70	MHET, TPA, EG	[87,88]
PE-H	*Pseudomonas aestusnigri*	6SBN	Amorphous PET film	30	MHET	[89]
PET2	*Uncultured bacterium from marine metagenome*	C3RYL0	PET nanoparticle agar	50	TPA, zone of clearance	[90]
PET5	*Oleispira antarctica RB-8*	R4YKL9	PET nanoparticle agar	50	Zone of clearance	[90]
PET6	*Vibrio gazogenes*	UPI0003945E1F	PET nanoparticle agar	50	Zone of clearance	[90]
PET12	*Polyangium brachysporum*	A0A0G3BI90	PET nanoparticle agar	50	Zone of clearance	[90]
PmC	*Pseudomonas mendocina*	-				
Tcur0390	*Thermomonospora curvata DSM 43183*	CDN67546.1	PET nanoparticle suspension	50	Reduced turbidity	[73]
Tcur1278	*Thermomonospora curvata DSM 43183*	CDN67545.1	PET nanoparticle suspension	60	Reduced turbidity	[73]
Tfca	*Thermobifida fusca* KW3	FN401519.1	Cyclic PET trimers	50–60	MHET, BHET	[91]
TfCut1	*Thermobifida fusca KW3*	CBY05529.1	PET film	55–65	≥12% weight loss	[92]
TfCut2	*Thermobifida fusca KW3*	CBY05530.1	PET film	55–65	≥12% weight loss	[92]
TfH	*Thermobifida fusca DSM43793*	WP_011291330.1	Bottle-grade PET (10% crystallinity)	55	≈50% weight loss	[93]
Tha_Cut1	*Thermobifida alba DSM43185*	ADV92525.1	3PET	50	TPA, HEB, MHET	[94]
Thc_Cut1	*Thermobifida cellulosilytica*	ADV92526.1	3PET and PET film (37% crystallinity)	50	TPA, HEB, MHET	[95]
Thc_Cut2	*Thermobifida cellulosilytica*	ADV92527.1	3PET and PET film (37% crystallinity)	50	TPA, HEB, MHET	[95]
Thf42_Cut1	*Thermobifida fusca DSM44342*	ADV92528.1	3PET and PET film (37% crystallinity)	50	TPA, HEB, MHET	[95]
Thh_Est	*Thermobifida halotolerans DSM 44931*	AFA45122.1	3PET	50	TPA, BA, HEB, MHET	[80]

3PET, bis(benzoyloxyethyl) terephthalate; TPA, terephthalic acid; BA, benzoic acid; EG, ethylene glycol; HEB, hydroxyethylbenzoate; MHET, mono-(2-hydroxyethyl) terephthalate; BHET, bis(2-hydroxyethyl) terephthalate; lcPET, low crystalline.

## Data Availability

Not applicable.
